# Estimation of Vitamin D Levels in Patients With Retinal Vein Occlusions and a Comparison With Age-Matched Control Groups

**DOI:** 10.7759/cureus.38909

**Published:** 2023-05-11

**Authors:** Varsha Kandambeth, Prachee Nagrale, Sachin Daigavane

**Affiliations:** 1 Department of Ophthalmology, Jawaharlal Nehru Medical College, Datta Meghe Institute of Higher Education and Research, Wardha, IND

**Keywords:** diabetes, hypertension, cardio vascular disease, vitamin d levels, retinal vein occlusions

## Abstract

Background

In the older population, retinal vein occlusion (RVO) is a major contributor to vision loss and blindness. RVO is the second most common form of retinal vascular disease, following diabetic retinopathy. On the other hand, there is a paucity of studies on vitamin D insufficiency and its influence on the causation of RVOs. The goal of this study is to demonstrate a link between vitamin D levels of individuals in rural India who have RVOs.

Methods

This study is a hospital-based prospective case-control study. All patients aged 18 years and above with RVO visiting the ophthalmology outpatient department at a tertiary care facility in central India and all controls in the same age group were chosen for the study after taking into consideration inclusion and exclusion criteria. Fasting for 12 hours prior to blood sample collection was required of all participants. The total vitamin D concentration in the serum was determined using tandem mass spectrometry after it had been frozen at 20°C. For this study, vitamin D levels were collected from 70 participants.

Results

The average age is 60, with a standard variation of 10 for both cases and controls. There is a 49% prevalence of central RVO (CRVO), 34% prevalence of inferotemporal branched RVO (IT BRVO), and 17% prevalence of superotemporal BRVO (ST BRVO). Twenty percent of the 35 patients were deficient in vitamin D, and 80% had insufficient levels. No case patient had vitamin D levels within the normal range. No one with vitamin D insufficiency was found among the 35 controls. Twenty-five percent of the patients had adequate vitamin D levels, but only 28.6% of the controls did. The p-value of 0.01 indicates a remarkable difference in vitamin D levels between the diagnosed cases and controls. Cases had mean vitamin D levels of 21.408 +/− 4.947 ng/dl, while controls had mean levels of 37.808 +/− 11.799 ng/dl. Vitamin D levels did not differ significantly across RVO subtypes. The study also shows the association of RVO with hypertension (HTN) and dyslipidemia as the p-value was noted to be significant (p = 0.0147 < p = 0.05) for HTN with an odds ratio of 3.43 (CI, 1.25-9.4) and was significant (p = 0.0404 < p = 0.05) for dyslipidemia with an odds ratio of 4.87 (CI, 0.96-24.97). Diabetes, smoking, hyperhomocysteinemia, dyslipidemia, cardiovascular disease, and cerebrovascular accident are all well-known risk factors, but we found no evidence associating them together.

Conclusion

Vitamin D proved to be an important risk factor in the causation of RVOs. Other risk factors like HTN and dyslipidemia also showed significant relation in the study. Vitamin D levels should be advised as a routine investigation in patients who are diagnosed with RVOs along with screening for other risk factors. Vitamin D supplementation should be given as prophylaxis in cases of deficiency.

## Introduction

In the elderly population, retinal vein occlusions (RVOs) are the second most important cause of vision loss due to vascular disease, following closely behind diabetic retinopathy as the most common cause [1]. RVOs are divided into three categories: hemicentral RVO (also known as HCRVO), branch RVO (also known as BRVO), and central RVO (CRVO). These categories are determined by the location of the blockage. Rogers et al. [2] came to their conclusions regarding the prevalence of RVO all over the world by combining data on 68,751 individuals that were collected throughout research projects carried out in the United States, Europe, Asia, and Australia. They determined that the overall frequency of RVO was 5.20 per 1,000 people by using age and gender as controls. They found that the prevalence of branch RVOs (BRVOs) was 4.42, while the prevalence of CRVOs was 0.80. Approximately 0.8% of adults had RVOs, according to a population-based study by Jonas et al. in rural Central India; however, BRVOs were found to be about seven times more common than CRVOs [3]. The intricate etiology of RVO is still not fully understood. Multiple large studies using hospital-based controls have found that patients who have systemic arteriosclerotic vascular diseases have a significantly increased risk of RVO. The relationship between RVO, cardiovascular disease (CVD), and stroke has been the subject of multiple articles [4]. Additional key risk factors include advancing age, hypertension (HTN), dyslipidemia, diabetes, and hyperhomocysteinemia [5].

Vitamin D, a fat-soluble vitamin, can be obtained from food or synthesized from 7-dehydroxycholesterol in the body’s subcutaneous fat in response to subjection to ultraviolet rays. Vitamin D is crucial for strong teeth and bones. Researchers have noted a link between vitamin D insufficiency and a high risk of ischemic stroke, coronary artery disease (CAD), venous thromboembolism, and death [6]. Vitamin D deficiency has been related to a high risk of death from cardiovascular and cerebrovascular diseases in large population studies [7]. There has been a lot of research done on the impact that vitamin D has on the endothelium of diabetics, and the results have been quite informative. Supplementation with vitamin D improved the function of the vascular endothelium [8]. It is necessary to do additional research to validate the probable role that vitamin D deficiency plays on the retinal vascular endothelium that may result in the development of RVO. The findings of a significant study conducted on individuals suffering from heart failure and vitamin D indicate that treatment with vitamin D improves outcomes [9]. Research on patients with type 2 diabetes and ischemic heart disease is looking at the influence that vitamin D has on the vascular endothelium and how vitamin D therapy can enhance endothelial function. These positive findings of the research support the hypothesis that vitamin D may be involved in the retinal vasculature.

We have a hypothesis that vitamin D may be involved in the etiology of RVOs as vascular endothelial function improves with vitamin D supplementation, and the risk factors for RVO and other vascular diseases share similar pathophysiology of atherosclerosis.

## Materials and methods

After meeting the inclusion and exclusion requirements, 70 participants in all were registered for this case-control hospital-based study. Thirty-five of them were selected as cases after being confirmed with RVO. Thirty-five individuals in the matching age range as the cases who have been confirmed to be free of RVO were taken as controls. The Datta Meghe Institute of Higher Education and Research’s Institutional Ethical Committee (IEC) gave approval (IEC/2020-21/9361), and the study was carried out in accordance with the Helsinki Declaration.

Each patient was given complete and accurate information regarding the procedure. Patients who met the trial’s requirements were enrolled one by one in the study. A detailed patient history was taken and recorded. Medical records were used to corroborate the presence of a patient’s reported history of diabetes mellitus (DM), HTN, dyslipidemia, CAD, or cerebrovascular accident (CVA).

General examinations including pulse, peripheral pulses, blood pressure, and higher functional status were also noted. Comprehensive ophthalmic examinations, including best-corrected visual acuity (Snellen chart), slit lamp examination, intraocular pressure (IOP) measurement by applanation tonometer, and fundoscopy (slit lamp biomicroscopy with 90 D), were performed on all patients. Fundus photographs were taken on the retinal camera (Visucam 524, Carl Zeiss Meditec AG, Dublin, CA) as documentation. In case there was any suspicion of macular edema, optical coherence tomography (Cirrus HD OCT, Carl Zeiss Meditec AG) was obtained. Patients had their blood pressure taken, as well as fasting and postprandial blood sugar, lipid profiles, homocysteine levels, and electrocardiograms (ECGs), as part of a comprehensive evaluation of their systems.

Fasting for 12 hours prior to blood sample collection was required of all participants. The total vitamin D concentration in the serum was determined using tandem mass spectrometry after it had been frozen at 20°C. For this study, vitamin D levels were collected from 70 participants. Deficient levels are deemed to be below 20 ng/ml, whereas insufficient levels are between 20 and 30 ng/ml.

Inclusion criteria

Subjects older than 18 years were selected for the study; patients were chosen without regard to sex; subjects were chosen after obtaining their informed written consent, which was translated into the patients’ native language.

Exclusion criteria

Vitamin D-supplemented patients under the age of 18, patients on special diets prescribed by a doctor, and participants with renal disease, hepatic disease, skin disease, or persistent alcoholism were not allowed to participate in the study.

## Results

**Figure 1:**
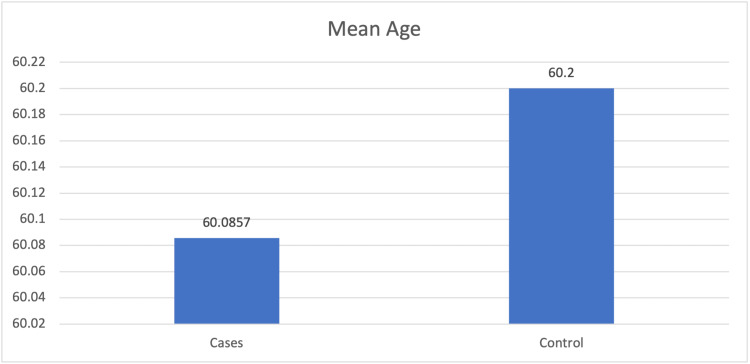
Mean age of cases and controls in years

Out of 70 subjects included in the study, 35 are cases of RVO and 35 are controls. The mean age group in cases is 60.085 +/− 10.842 and controls is 60.2 +/− 10.856. Between cases and controls, the age group did not significantly differ. The minimum age observed was 30 years in both cases and controls, whereas the maximum age was 80 years in cases and 84 years in controls (Figure 1). The genders of cases and controls did not significantly differ from one another (p = 0.47).

**Figure 2:**
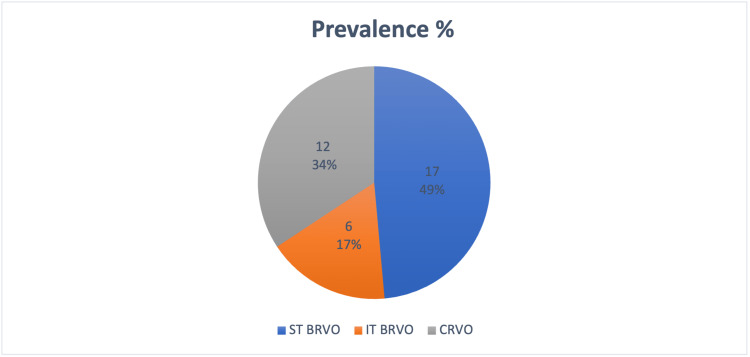
Prevalence of different types of retinal vein occlusions ST BRVO, superotemporal branched retinal vein occlusion; IT BRVO, inferotemporal branched retinal vein occlusion; CRVO, central retinal vein occlusion

Regarding the prevalence of different forms of RVO among the cases, 17 out of 35 patients or 49% of the cases had ST BRVO. Twelve patients (or 34% of the total) had CRVO, while six (or 17% of the total) had inferotemporal branched RVO (IT BRVO) (Figure 2).

**Figure 3:**
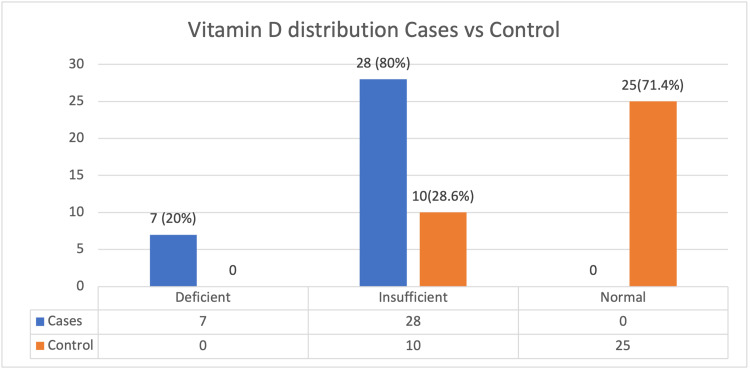
Vitamin D levels between cases and controls

Seven patients (20%) out of the 35 cases had deficient levels of vitamin D or less than 20 ng/ml. Vitamin D levels in 28 individuals (80%) were between 20 and 30 ng/ml, which is considered to be insufficient. Vitamin D levels were abnormal in all of the cases. Out of the 35 controls, there was no one with deficient levels of vitamin D. In all, 10 (28.6%) subjects among the controls had insufficient levels of vitamin D, and 25 (71.4%) patients had normal levels of vitamin D. The p-value was found to be 0.01 < 0.05, which is significant (Table 1, Figure 3).

**Table 1:** Vitamin D levels between cases and controls

	Cases		Controls			
	Frequency	Percent	Frequency	Percent	Chi-square	p-value
Deficient	7	20	0	0	40.52	<0.01*
Insufficient	28	80	10	28.6		
Normal	0	0	25	71.4		
Total	35	100	35	100		

The average vitamin D levels in patients with different types of RVOs were found to be as follows: 20.8667 in those with CRVO, 21.9235 in those with ST BRVO, and 21.0333 in those with IT BRVO. Given that the p-value was 0.84 > 0.05 (Table 2), there was no statistically significant difference between the various forms of RVOs and vitamin D levels (Table 2).

**Table 2:** Vitamin D levels in different types of RVOs RVO, retinal vein occlusion; ST BRVO, superotemporal branched retinal vein occlusion; IT BRVO, inferotemporal branched retinal vein occlusion; CRVO, central retinal vein occlusion

RVO type	Frequency	Mean	Standard deviation	Standard error	Lower bound	Upper bound	Minimum	Maximum	F-value	p-value
ST BRVO	17	21.9235	5.31572	1.28925	19.1904	24.6566	11.50	29.00	0.172	0.842 (95% CI)
IT BRVO	6	21.0333	2.09539	0.85544	18.8344	23.2323	17.20	23.20		
CRVO	12	20.8667	5.63614	1.62701	17.2856	24.4477	8.00	25.20		
Total	35	21.4086	4.94787	0.83634	19.7089	23.1082	8.00	29.00		

Out of all the cases, the number of hypertensives in cases was 26 (74.2%) compared to 16 (45.7%) in controls. The odds ratio is 3.43 (CI, 1.25-9.4), which suggests that hypertensives have a higher risk of RVO (Table 3).

**Table 3:** Prevalence of hypertension in cases and controls

Hypertension		Cases	Controls	Total	Chi-square	p-value	Odds ratio
Yes	Frequency	26	16	38	5.9524	0.0147 *S	3.43 CI (1.25-9.4)
	%	74.29%	45.70%	54.30%			
No	Frequency	9	19	32			
	%	25.71%	54.30%	45.70%			
Total	Frequency	35	35	70			
	%	100%	100%	100%			

Out of all the cases, the number of dyslipidemia in cases was eight (22.86%) compared to two (5.72%) in controls. The odds ratio is 4.89 (CI, 0.96-24.97), which suggests that subjects with dyslipidemia might have a higher risk of RVO (Table 4).

**Table 4:** Prevalence of dyslipidemia in cases and controls

Dyslipidemia		Cases	Controls	Total	Chi-square	p-value	Odd ratio
Yes	Frequency	8	2	10	4.2	0.0404*S	4.89 CI (0.96-24.97)
	%	22.86%	5.72%	14.28%			
No	Frequency	27	33	60			
	%	77.14%	94.28%	85.72%			
Total	Frequency	35	35	70			
	%	100%	100%	100%			

HTN and dyslipidemia proved a significant risk factor in cases compared to diabetes, CVA or CAD, hyperhomocysteinemia, and smoking, which were all previously established risk factors (Table 5).

**Table 5:** Comparison of different risk factors for retinal vein occlusions CVA, cerebrovascular accident; CAD, coronary artery disease

Parameter	Cases	Controls
Hypertension	74.29%	45.70%
Dyslipidemia	22.86%	5.72%
Diabetes	22.9%	22.9%
Smoking	5.7%	0.0%
CVA/CAD	28.6%	31.4%
Hyperhomocysteinemia	6%	Not estimated

Since HTN and dyslipidemia showed risk factors in our study, we tried to obtain a relationship between the vitamin D levels in cases with HTN and dyslipidemia but could not obtain any significant relationship between the two. The hypertensive group had a mean vitamin D value of 20.63 +/− 5.11 compared to the non-hypertensive group with a vitamin D mean value of 23.58889 with a standard deviation of 3.90271 and a mean difference of 1.566. A p-value of 0.127 was obtained and was found to be insignificant. Similarly, while comparing the relation between vitamin D levels and dyslipidemia, it was found that the dyslipidemia group recorded a vitamin D mean value of 21.4500 with a standard deviation of 6.21128 compared to subjects with no history of dyslipidemia with a vitamin D mean value of 21.3963 with a standard deviation of 4.65043. With a mean difference of 0.537, it was found to be insignificant as the p-value was 0.979.

## Discussion

One of the most frequent reasons for an abrupt, unilateral diminution of vision, particularly in older age groups, is RVO. The incidence of RVO is caused by a number of risk factors. The precise pathophysiology has not yet been determined. The main risk factor for RVO is age. Blood hyperviscosity, thrombophilia, diabetes, heart disease, vascular cerebral stroke, and arteriosclerosis are additional risk factors. RVO has a close relationship to metabolic syndrome. Black race; congenital thrombophilic disorders such as hyperhomocysteinemia, factor V Leiden mutation, and anticardiolipin antibodies; and end-organ damage from DM and HTN are all associated with a much greater chance of developing RVO. Smoking, Behcet disease, and chronic systemic inflammatory illnesses, such as vasculitis, all raise the risk. Glaucoma and ocular HTN are ophthalmic risk factors for RVO. All of these are known risk factors for RVO. These elements are covered in depth in a meta-analysis by Kolar [10]. Less research has been done on the link between vitamin D and a risk factor for RVO. In contrast to controls, the majority of whom had adequate vitamin D levels, all patients with RVO in our study had deficient or insufficient vitamin D levels. Deficient levels of vitamin D are those below 20 ng/ml [11]. Values above 30 ng/ml are regarded as normal, while values between 20 and 30 ng/ml are regarded as insufficient [12]. In our study, 20% of cases had vitamin D deficiency and 80% had insufficient levels, showing that all cases were either deficient or had insufficient amounts of vitamin D, while 71.4% of controls had normal levels. As a result, the levels of vitamin D between the patients and controls differ significantly.

According to Oli and Joshi's case-control study in a south Indian population, 95% of patients with RVOs had vitamin D levels below 20 ng/ml, compared to just 8% of controls [13]. This finding was favorable to our research. This supports the current study, where vitamin D levels were low in 20% of cases and high in 80% of patients. Epstein et al.'s investigation also discovered considerably lower vitamin D levels in cases compared to controls, but they were unable to detect any discernible difference in vitamin D levels between cases and controls [14]. In patients with retinal vascular blockage, a case report by Talcott and Eliott also revealed severe vitamin D deficiency [15]. According to research by Karimi et al., patients receiving oral supplements had higher reductions in central macular thickness and better improvements in best-corrected visual acuity after undergoing intravitreal bevacizumab therapy [16].

A peculiar case of bilateral central retinal vein blockage in a patient with only vitamin D insufficiency and no other known systemic risk factors was also discovered in the present study. The patient improved drastically after the bevacizumab injection and oral vitamin D supplementation. The current study also compared the levels of vitamin D in different types of RVO, but no notable difference was found in either. Hypertensives and people with dyslipidemia had a higher risk of getting the condition and thus demonstrated a strong relationship when considering the other risk factors connected to RVO. However, in our study, the already known risk factors like diabetes, smoking, hyperhomocysteinemia, CVD, and CVA did not show any significant relation. Our study is favored by O’Mahoney's meta-analysis, where HTN and high cholesterol are thought to have a higher risk of RVO, while adults with DM are thought to have a lower risk of the condition [17]. Dodson et al. observed that HTN and hyperlipidemia are the most common underlying medical disorders causing RVO even in diabetics and that these may play an important role in the etiology as they do in the non-diabetic, which coincided with our findings [18]. Our attempt to find out any relationship between HTN and dyslipidemia with the amount of vitamin D was found to be unfruitful.

Hence, all patients with vitamin D deficiency were also commenced on oral vitamin D treatment. The dosage of vitamin D supplementation that should be taken is still a subject of study; hence, up to a maximum dose of 4000 IU/day is considered advisable [19]. But the limitations of our study include that vitamin D levels were not estimated at the time of RVO, making it unable to rule out the likelihood of a delayed onset vitamin D deficiency; it is necessary to conduct larger, randomized controlled trials to ascertain whether vitamin D supplementation can control RVO; and seasonal variations in vitamin D levels were not taken into consideration. Vitamin D mainly acts on the endothelium. Kim et al. talk in detail about the effect of vitamin D on endothelial function and the various mechanisms associated with it [20]. The vascular endothelium contains vitamin D receptors (VDRs) and 1-alpha-hydroxylase, which transform vitamin D into its active form [21]. Nitric oxide (NO), which is essential for maintaining vascular tone in healthy vascular physiology, inhibits oxidative activities that could be detrimental to the endothelium, as described earlier. Studies conducted in vitro have demonstrated that the relationship between vitamin D and VDR boosts NO production, lowering endothelial oxidative stress [22].

## Conclusions

RVOs are a prime cause of vision loss in the aged population. Hence, it is crucial to identify and manage the risk factors associated with them. In our study, low levels of vitamin D were found in all cases of RVO in comparison to age-matched control groups, proving our hypothesis valid. RVOs are closely related to multiple risk factors like HTN, dyslipidemia, diabetes, and atherosclerotic diseases, but their association with vitamin D is less explored. In this study, vitamin D proved to be a compelling risk factor in the causation of RVOs. Other risk factors like HTN and dyslipidemia also showed a notable relationship in the study. Hence, vitamin D levels should be advised as a routine investigation in individuals with RVOs, along with screening for other risk factors. Any patient with vitamin D deficiency should also be advised to undergo an ophthalmological examination to rule out any ocular pathologies. Vitamin D supplementation should be given as prophylaxis in cases of deficiency.
